# Identification of a Heme Activation Site on the MD-2/TLR4 Complex

**DOI:** 10.3389/fimmu.2020.01370

**Published:** 2020-06-30

**Authors:** John D. Belcher, Ping Zhang, Julia Nguyen, Zachary M. Kiser, Karl A. Nath, Jianjun Hu, John O. Trent, Gregory M. Vercellotti

**Affiliations:** ^1^Division of Hematology, Oncology and Transplantation, Department of Medicine, University of Minnesota, Minneapolis, MN, United States; ^2^Division of Nephrology and Hypertension, Mayo Clinic, Rochester, MN, United States; ^3^College of Engineering and Computing, University of South Carolina, Columbia, SC, United States; ^4^Departments of Medicine, and Biochemistry and Molecular Genetics, James Graham Brown Cancer Center, University of Louisville, Louisville, KY, United States

**Keywords:** MD-2, TLR4, heme, LPS, NF-κB

## Abstract

Myeloid differentiation factor-2 (MD-2) binds lipopolysaccharide (LPS) and initiates toll-like receptor-4 (TLR4) pro-inflammatory signaling. Heme also activates TLR4 signaling, but it is unknown if heme interacts with MD-2. Therefore, we examined MD-2 for a potential heme activation site. Heme-agarose and biotin-heme/streptavidin-agarose pulled down recombinant MD-2, which was inhibited by excess free heme. UV/visible spectroscopy confirmed MD-2-heme binding. To determine whether MD-2 was required for heme-mediated TLR4 signaling, HEK293 cells were transfected with MD-2, TLR4, CD14, and an NF-κB luciferase reporter, and then stimulated with heme or LPS. Heme or LPS treatment elicited robust reporter activity. Absence of MD-2, TLR4 or CD14 plasmid abolished NF-κB reporter responses to heme or LPS. *In silico* analysis identified two potential heme docking sites on MD-2 near conserved amino acids W23/S33/Y34 and Y36/C37/I44. Heme-induced NF-κB activity was reduced by 39 and 78% in HEK293 cells transfected with MD-2 mutants W23A and Y34A, respectively, compared to WT-MD-2. NF-κB activation by LPS was not affected by the same mutants. Biotinyl-heme/streptavidin-agarose pulled down 68% less W23A and 80% less W23A/S33A/Y34A mutant MD-2 than WT-MD-2. In contrast, at the Y36/C37/I44 MD-2 site, heme-induced NF-κB activity was significantly increased by mutants Y36A (191% of WT-MD-2) and unchanged by mutants C37A and I44A (95 and 92%, respectively, of WT-MD-2). In conclusion, these data suggest that heme binds and activates TLR4 signaling at amino acids W23 and Y34 on MD-2.

## Introduction

Toll-like receptors are central to vertebrate innate immune responses (1, 2). They recognize broad but highly conserved structural patterns on bacteria, fungi and viruses called pathogen-associated molecular patterns or PAMPs as well as non-pathogenic chemicals and non-patterned molecules. Unique among toll-like receptors, TLR4 activity depends on a molecular interaction with the extracellular adaptor protein MD-2 (3, 4). TLR4 and MD-2 form a heterodimer that recognizes LPS molecules delivered to MD-2/TLR4 by CD14 (5). LPS binds to a large hydrophobic pocket in MD-2 and directly bridges the MD-2/TLR4 heterodimer (6). Binding of LPS to MD-2 triggers homodimerization of MD-2/TLR4 complexes and recruitment of specific adaptor proteins to TLR4 cytoplasmic domains that initiates a signaling cascade leading to the activation of NF-κB, inflammasome formation, and production of pro-inflammatory cytokines such as TNF-α, IL-1β, IL-8, and IL-18 by macrophages, and the expression of adhesion molecules such as P-selectin and von Willebrand factor on endothelium (7–11).

The MD-2/TLR4 complex also recognizes a diverse number of endogenous molecules released from injured cells called damage-associated molecular patterns or DAMPs. One such DAMP is heme (7, 9, 12). The activation of MD-2/TLR4 by heme is distinct from the activation of MD-2/TLR4 by LPS (7). An anti-TLR4/MD2 antibody or a lipid A antagonist inhibits LPS-induced, but not heme-induced MD-2/TLR4 signaling and conversely, protoporphyrin IX inhibits heme-induced, but not LPS-induced MD-2/TLR4 signaling (7, 9).

Large amounts of heme can be released intravascularly by trauma, sepsis, malaria and red blood cell disorders such as sickle cell disease (SCD). Recent studies underscore the importance of heme-mediated MD-2/TLR4 activation in inflammation, vaso-occlusion, lethality and pulmonary injury in SCD (9, 13). In monocyte/macrophages, heme promotes a pro-inflammatory M1 phenotype, induces tissue factor, and activates coagulation in a TLR4-dependent fashion (9, 14, 15). We previously demonstrated that TLR4 signaling is required for vaso-occlusion induced by heme in SCD mice (9). In endothelial cells, heme mobilizes Weibel-Palade body P-selectin and von Willebrand factor onto the cell surface within minutes, activates the pro-inflammatory transcription factor NF-κB, and induces microvascular stasis in SCD mice in a TLR4-dependent manner. All of these heme-mediated effects can be blocked by adding back the high-affinity heme scavenger hemopexin that is depleted in the plasma of SCD mice and patients (9, 16). Disrupting heme-mediated MD-2/TLR4 signaling might provide a potential therapeutic opportunity to interrupt heme-mediated inflammation and vaso-occlusion in SCD and other hemolytic conditions. Because of heme and MD-2's mutual hydrophobicity and the precedence for LPS binding to MD-2, we explored potential heme activation sites on MD-2.

## Materials and Methods

### Site-Directed Mutagenesis of Human MD-2

Plasmid pFlag-CMV1–hMD2 was a gift from Doug Golenbock (Addgene plasmid #13028). Site-directed mutagenesis was performed using a QuickChange II XL site-directed mutagenesis kit (Agilent Technologies). Mutagenic primers were designed using web-based QuickChange primer design program, and synthesized by Integrated DNA Technologies (IDT) with polyacrylamide gel electrophoresis (PAGE) purification. Mutant strands were synthesized and transformed into XL10-gold ultracompetent cells (Agilent), the DNA from the colonies were checked with restriction digestion and confirmed with DNA sequencing.

### Expression and Purification of N-Flag Tagged Recombinant MD-2 and Its Mutants

WT and mutant N-Flag-hMD-2 fragments from pFlag-CMV1–hMD-2 plasmids were subcloned into expression plasmids with a CAG promoter (pT2/Caggs-Flag-hMD2) that were used to produce WT and mutant MD-2 recombinant proteins in Chinese Hamster Ovary Cells (CHO) as described previously (16) with modifications. CHO cells were maintained in T225 flasks in RPMI-1640 with L-glutamine (Gibco) supplemented with 10% fetal bovine serum (FBS) in a 5% CO_2_ incubator at 37°C. Cells in T225 cm^2^ culture flasks were transiently transfected with polyethylenimine (PEI, linear, MW 2500) (Polysciences) using a 4:1 ratio of PEI to DNA (w/w). After 18 h at 37°C, the cells were changed to ProCHO-AT protein-free media (Lonza). After 4 days in ProCHO-AT media, the conditioned media was collected, and cleared by centrifugation at 600× g for 30 min at 4°C and filtered using a 0.22 μm Stericup Vacuum Filtration System (EMD Millipore). In the UV/Vis heme-MD-2 binding assays described below, the recombinant proteins in the filtered media were purified using anti-Flag M2 affinity gel (Sigma-Aldrich) column chromatography following the manufacturer's instructions. The bound Flag fusion proteins were eluted by competition with Flag peptide, and further concentrated using 10k centrifugal filter units (Amicon). The purity and concentration of the protein was determined by a 4–15% SDS-PAGE and Coomassie R-250 stain (Bio-Rad) with BSA standards (0.2–5 μg) loaded on the same gel to estimate the Flag-MD-2 concentration by comparing the band intensities with BSA. The recombinant WT and mutant MD-2 proteins were confirmed by Western blots with a primary MD-2 antibody (Abcam).

### Heme-Agarose and Biotin-Heme/Streptavidin-Agarose Pull-Down Assays

Pull-down assays were used to determine if there was a physical interaction between MD-2 and heme. Conditioned ProCHO-AT culture media (30 ml) from T225 tissue culture flasks containing CHO cells overexpressing recombinant wild-type (WT) Flag-MD2, mutants or recombinant Flag-hemopexin (Hpx) as a positive control as previously described (16) were concentrated to 3 ml using a 10k centrifugal filter units (Amicon). For the heme-agarose pull-down assays, 1 ml of the concentrated ProCHO-AT media was incubated overnight at 4°C with 35 μl heme-agarose, or control agarose beads (Sigma, pre-washed with PBS). For the biotin-heme/streptavidin-agarose pull-down assays, 1 ml of concentrated ProCHO-AT media was incubated with 15 μM biotin-heme (Frontier Scientific) overnight at 4°C in the dark, then 40 μl of 50% streptavidin-agarose (Sigma-Aldrich) was added to the mixture and incubated an additional 2 h at 4°C. To test the binding specificity of biotin-heme and recombinant MD-2 proteins, 1 ml of concentrated ProCHO-AT media was pre-incubated with a 6.7-fold excess of unlabeled free heme (100 μM) for 2 h at 4°C before the incubation with 15 μM biotin-heme. The resulting beads from both heme-agarose and streptavidin-agarose pull-down assays were washed with PBS 6 times and run on an SDS-PAGE Western bot using an anti-Flag monoclonal antibody (Sigma-Aldrich) for detection. Hematin, herein referred to as heme, was prepared immediately before use by mixing 10 mg hemin chloride (Frontier Scientific), 10 mg D-sorbitol (Sigma-Aldrich), and 6.9 mg sodium carbonate (Sigma-Aldrich) in 5.7 ml of sterile saline (Baxter) for 30 min in the dark. All heme preparations, appropriately diluted in saline, were filtered at 0.22 μm before use. Endotoxin levels were monitored using a Limulus amebocyte lysate test (GenScript). Heme preparations contained <0.01 endotoxin units/ml at 10 mM heme; all assays used 10 μM heme.

### UV/Vis Heme-MD-2 Binding Assays

The UV/Vis absorption spectra (250–600 nm) of heme, MD-2 and heme + MD-2 were measured using a Nanophotometer P330 (Implen) with an optical path length of 1 cm. Human recombinant Flag-Hpx was used as a positive heme-binding control.

### *In situ* Identification of Potential Heme-Binding Sites on MD-2

Potential heme-binding amino acid (AA) residues on human and mouse MD-2 were identified using the HemeBind web server that is freely accessible online (http://mleg.cse.sc.edu/hemeBIND/). HemeBind is a specialized algorithm that combines both structure- and sequence-based methods to identify potential heme-binding sites on heme proteins (17).

### NF-κB Reporter Assays

Human embryonic kidney 293 (HEK293) cells were seeded in 96-well plates at 2 × 10^4^ cells per well and incubated overnight in a 5% CO_2_ incubator at 37°C. In the morning, cells were transiently transfected with 50 ng TLR4 expression vector (pcDNA3.1-hTLR4, a gift from Ruslan Medzhitov, Addgene plasmid #13086) or empty vector, along with 15 ng of *Firefly* luciferase NF-κB reporter vector (pNifty-Luc, InvivoGen), 15 ng of *Renilla* luciferase control vector (pRL-TK, Promega), 10 ng of wt or mutant MD-2 expression vector (pFlag-CMV1-hMD2), and 10 ng of CD14 expression vector (pCDNA3.1-hCD14, a gift from Doug Golenbock, Addgene plasmid #13645) per well using Lipofectamine Plus reagent (LifeTechnology). Four h after transfection, cells were incubated 24 h in media containing 10% fetal bovine serum (FBS). Twenty-four hours after transfection, cells were incubated with media with 1% FBS (control), or media with 1% FBS supplemented with heme (10 μM), LPS (10 ng/ml, Escherichia coli, serotype O111:B4; Sigma-Aldrich) or heme + LPS for 6 h, then cells were lysed and luciferase activity was measured.

### Statistical Analysis

Results are presented as means ± standard deviation unless otherwise indicated. Analyses were performed with SigmaStat 3.5 for Windows (Systat Software, San Jose, CA). Comparisons of multiple treatment groups were made using One Way ANOVA with Holm-Sidac correction, Kruskal-Wallis One Way Analysis of Variance on Ranks, or a student's unpaired *t*-test. Statistical significance was considered to be *p* < 0.05.

## Results

### Heme Binding to MD-2

To determine if heme binds to MD-2, recombinant MD-2 was expressed by transfecting CHO cells with a plasmid encoding human MD-2 with a Flag tag at the N-terminus. After transfection, cells were washed and incubated for 3 or 4 days in protein-free ProCHO medium. Transfected, but not untransfected CHO cells secrete soluble recombinant human MD-2 into the media (Supplemental Figure 1). Flag-MD-2 had an apparent molecular weight of ~25 kDa, which is close to the predicted mass of 19.2 kDa.

**Figure 1:**
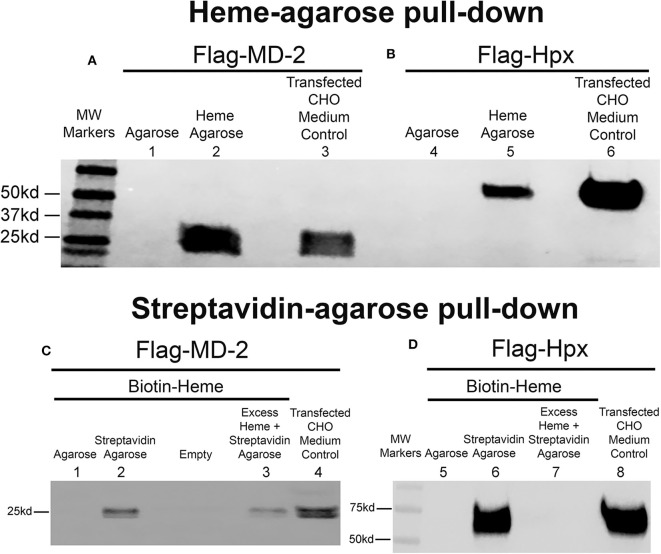
Heme binds to MD-2 in pull-down assays. Recombinant MD-2 and hemopexin (Hpx) were expressed by transfecting Chinese hamster ovary (CHO) cells with plasmids encoding human Flag-MD-2 or Flag-Hpx. Flag-Hpx served as a positive control for heme binding. After transfection, cells were washed and incubated for 72 h in protein-free CHO medium to allow the recombinant proteins to be transcribed, translated, and secreted into the CHO media. After 72 h, Flag-MD-2 (**A**, lane 3 and **C**, lane 4) and Flag-Hpx (**B**, lane 6 and **D**, lane 8) were present in the conditioned media of transfected CHO cells as demonstrated by a Western blot of the concentrated media with an anti-Flag primary antibody. **(A,B)** Heme-agarose pull-down assays. Conditioned CHO media containing **(A)** Flag-MD-2 or **(B)** Flag-Hpx were incubated overnight at 4°C with heme-agarose (lanes 2 and 5) or control agarose beads (lanes 1 and 4) and then pelleted, washed, and run on a Western blot with anti-Flag detection. **(C,D)** Streptavidin-agarose pull-down assays. Conditioned CHO media containing **(C)** Flag-MD-2 or **(D)** Flag-Hpx were incubated with 15 μM biotin-heme overnight at 4°C in the dark, then streptavidin-agarose (lanes 2, 3, 6, and 7) or control agarose (lanes 1 and 5) was added to the mixture for an additional 2 h at 4°C. After incubation, the agarose pellets were washed and run on Western blots with anti-Flag detection. To test the binding specificity of biotin-heme and recombinant Flag-MD-2 (**C**, lane 3) and Flag-Hpx (**D**, lane 7), conditioned CHO media were pre-incubated with an excess of unlabeled free heme (100 μM) for 2 h at 4°C before the incubation with 15 μM biotin-heme. The results shown are representative of four (**A** and **B**) and two (**C** and **D**) independent experiments, respectively.

The recombinant human flag-MD-2 (rhMD-2) was highly purified as seen on an SDS PAGE gel stained with Coomassie Brilliant Blue R-250 (Supplemental Figure 2). After 4 days, Flag-MD-2 was present in the conditioned medium of transfected CHO cells as demonstrated by a Western blot of the medium with an anti-Flag primary antibody (Figure 1A, lane 3). The conditioned CHO medium containing Flag-MD-2 was incubated with heme-agarose beads in a pull-down assay. Flag-MD-2 in the medium was pulled down with heme-agarose as demonstrated on a Western blot of the pull-down proteins with anti-Flag IgG (Figure 1A, lane 2). Flag-MD-2 was not pulled down from conditioned medium by control agarose without heme (Figure 1A, lane 1). As a positive control, recombinant human hemopexin (Hpx), which binds heme with high affinity, was expressed by transfecting CHO cells with a plasmid encoding Flag-labeled Hpx. After 4 days, Flag-Hpx was present in the conditioned medium of transfected CHO cells as demonstrated by a Western blot with anti-Flag IgG (Figure 1B, lane 6). Flag-Hpx had an apparent molecular weight of ~59 kd, which is the predicted mass. The medium containing Flag-Hpx was incubated with heme-agarose beads in a pull-down assay. Flag-Hpx in the medium was pulled down with heme-agarose as demonstrated on a Western blot of the pull-down proteins with anti-Flag IgG detection (Figure 1B, lane 5). Flag-Hpx was not pulled down from conditioned medium by control agarose without heme (Figure 1B, lane 4).

To confirm heme binding to MD-2, additional pull-down assays were run using recombinant MD-2, biotin-heme and streptavidin-agarose (Figure 1C). Flag-MD-2 was present in the conditioned CHO medium as demonstrated by a Western blot of the medium with an anti-Flag primary antibody (Figure 1C, lane 4). Conditioned CHO medium containing Flag-MD-2 was incubated with biotin-heme followed by streptavidin-agarose. Flag-MD-2 in the medium of conditioned CHO cells was pulled down with biotin-heme + streptavidin-agarose as demonstrated by a Western blot of the pull-down proteins with anti-Flag IgG (Figure 1C, lane 2). Biotin-heme binding to MD-2 was markedly diminished in the presence of a 6.7-fold excess of unlabeled free heme in the assay, demonstrating specific heme binding to MD-2 (Figure 1C, lane 3). Flag-MD-2 was not pulled down from conditioned medium by control agarose without streptavidin (Figure 1C, lane 1). As a positive control, CHO medium containing Flag-Hpx was incubated with biotin-heme + streptavidin-agarose. Flag-Hpx was present in the conditioned CHO medium as demonstrated by a Western blot with anti-Flag IgG (Figure 1D, lane 8). Flag-Hpx in the medium was pulled down with biotin-heme + streptavidin-agarose as demonstrated by a Western blot of the pull-down proteins with anti-Flag IgG (Figure 1D, lane 6). Biotin-heme binding to Hpx was eliminated in the presence of a 6.7-fold excess of unlabeled free heme in the assay, demonstrating specific heme binding to Hpx (Figure 1D, lane 7). Flag-Hpx was not pulled down from conditioned medium by agarose without streptavidin (Figure 1D, lane 5).

**Figure 2:**
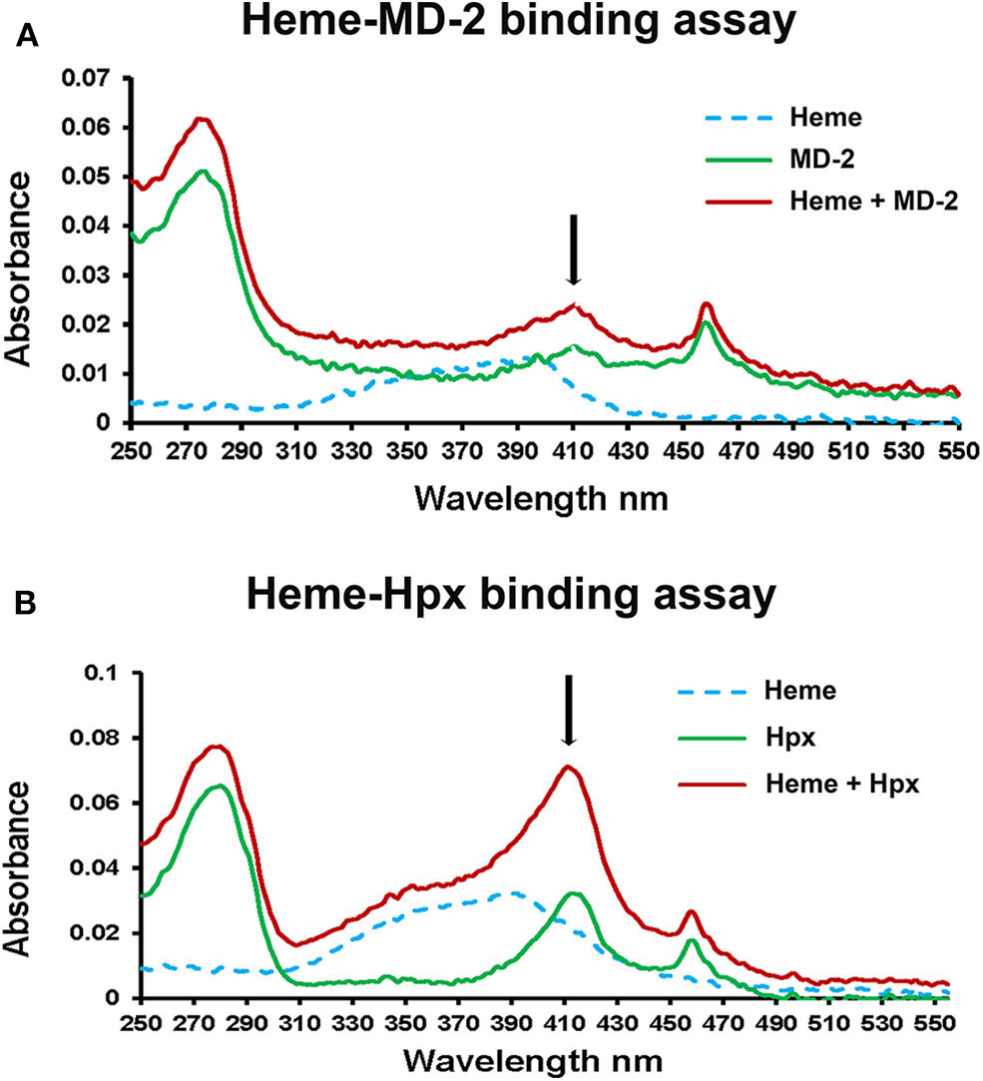
Heme binds to MD-2 in absorbance spectroscopy assay. **(A)** The UV/Vis absorption spectra (250–550 nm) of heme (10 μM, dashed blue line), recombinant MD-2 (green line), and heme + MD-2 (red line) were measured. **(B)** As a positive heme-binding control, the experiment was repeated using recombinant human Hpx instead of MD-2. The black arrows show the location of a characteristic Soret peak at ~414 nm seen in heme proteins. The results shown in **(A,B)** are representative of three independent experiments. Each spectrum was blanked against the buffer vehicle.

To further confirm heme-MD-2 binding, UV/visible spectroscopy was used to detect heme binding to MD-2. Proteins such as Hpx when bound to heme have an increase in absorbance at ~414 nm (18). Recombinant Flag-MD-2 and Flag-Hpx were purified from conditioned CHO media using anti-Flag-IgG-agarose affinity chromatography. A UV/visible absorbance scan of heme, Flag-MD-2, and Flag-MD-2 + heme demonstrated an increase in absorbance at 414 nm when heme was added to Flag-MD-2 (Figure 2A), consistent with heme binding to MD-2 that was similar to, but less than, Flag-Hpx + heme (Figure 2B).

### Heme Activation Sites on MD-2

**Figure 3:**
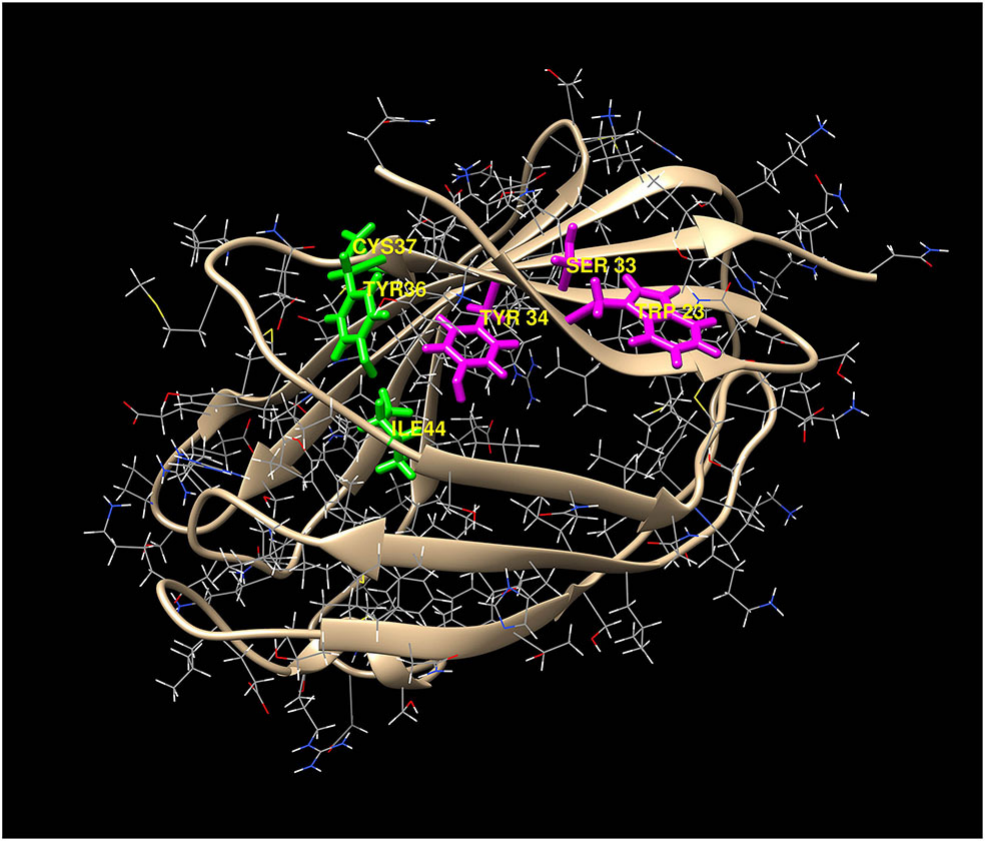
Two potential heme-docking sites on MD-2. Potential heme-binding amino acid residues on human and mouse MD-2 were identified using the online HemeBind algorithm that combines both structure- and sequence-based methods to identify potential heme-binding sites on heme proteins (17). These *in silico* analyses identified six potential heme binding amino acids located in two separate clusters on the surface of MD-2. The amino acids in the 2 clusters, W23/S33/Y34 (magenta) and Y36/C37/I44 (green), are highlighted on the 3-dimentional structure of human MD-2.

Potential heme-binding amino acid residues on human and mouse MD-2 were identified using the HemeBind web server that is freely accessible online. HemeBind is a specialized algorithm that combines both structure- and sequence-based methods to identify potential heme-binding sites on heme proteins (17). These *in silico* analyses identified two potential heme docking sites on both human and murine MD-2 near conserved amino acids W23/S33/Y34 and Y36/C37/I44 highlighted in magenta and green, respectively (Figure 3).

### NF-κB Reporter Assays With CD14, MD-2, and TLR4

**Figure 4:**
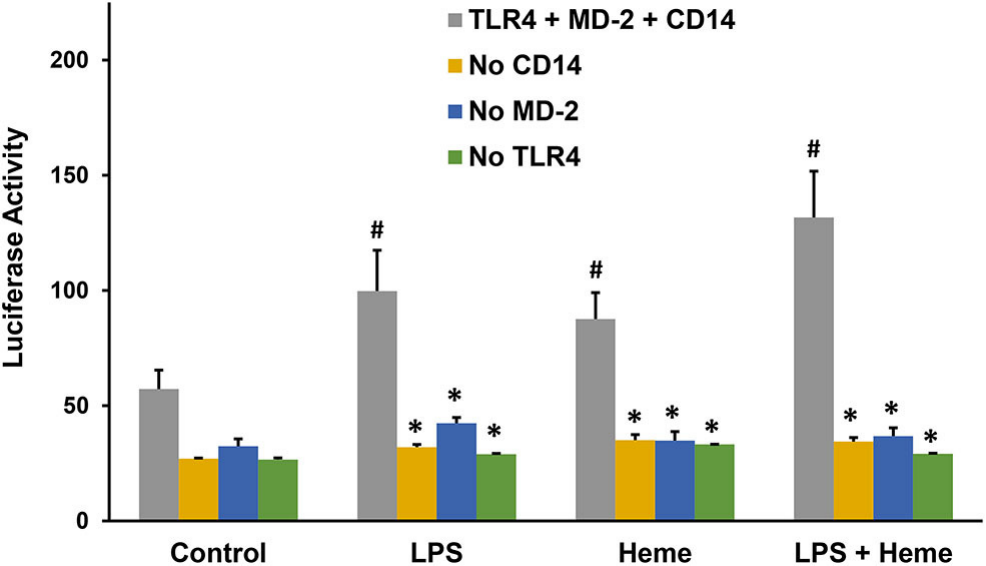
NF-κB Reporter Assays with TLR4, MD-2, and CD14. Human embryonic kidney 293 (HEK293) cells were transfected with plasmid expression vectors for *Firefly* NF-κB and *Renilla* luciferase reporters, plus CD14, MD-2, and TLR4 (gray bar); MD-2 and TLR4 (no CD14, orange bar); CD14 and TLR4 (no MD-2, blue bar); or CD14 and MD-2 (no TLR4, green bar). Twenty-four hours after transfection, cells were incubated with media (Control), 10 μM heme, 10 ng/ml LPS, or heme + LPS for 6 h in media containing 1% serum. After 6 h, cells were lysed and luciferase activity was measured and expressed as the ratio of *Firefly* luciferase NF-κB reporter to *Renilla* luciferase control. Results are representative of 3 independent experiments run in triplicate. Bars are means + SD. ^#^*P* < 0.01 control vs. heme, LPS, and heme + LPS. *P < 0.01 TLR4 + MD-2 + CD14 (gray bar) vs. no CD14 (purple bar), no MD-2 (orange bar), and no TLR4 (green bar) for each stimulant, using One Way ANOVA with Holm-Sidac correction.

LPS activation of TLR4 signaling requires CD14, MD-2 and TLR4, which leads to the activation of the pro-inflammatory transcription factor NF-κB. Previously, heme activation of TLR4 signaling in macrophages was shown to require TLR4 and CD14 (7), but it is unknown if heme activation of TLR4 requires MD-2. To determine whether heme-mediated TLR4 signaling requires MD-2, an NF-κB reporter assay was developed using HEK293 cells transfected with a *Firefly* luciferase NF-κB reporter with or without plasmids encoding MD-2, TLR4, and CD14. Twenty-four hours after transfection, HEK293 cells were incubated with media (control), heme (10 μM), LPS (10 ng/ml), or heme + LPS for 6 h, followed by measurement of NF-κB luciferase reporter activity. Heme, LPS and heme + LPS treatment elicited robust luciferase activity in the presence of CD14, MD-2, and TLR4 (Figure 4, gray bars). The absence of a CD14 (orange bars), MD-2 (blue bars) or TLR4 (green bars) inhibited NF-κB luciferase reporter responses to heme, LPS, and heme + LPS similar to control cells. TLR4, MD-2 and CD14 replete cells (gray bars) stimulated with heme + LPS had more activity than replete cells treated with heme or LPS alone.

### NF-κB Reporter Assays With WT or Mutant MD-2

**Figure 5:**
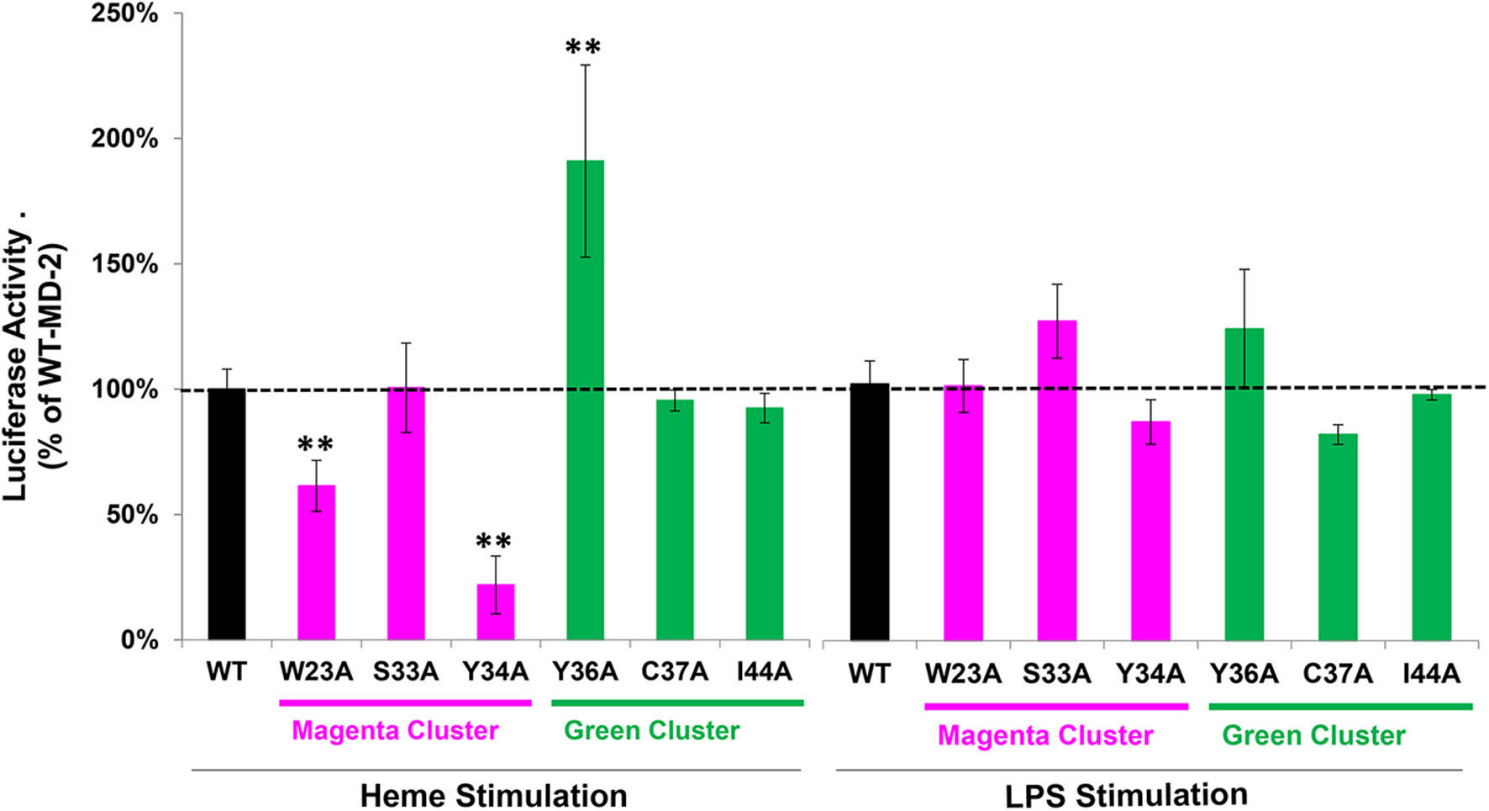
NF-κB reporter assays with WT and mutant MD-2. HEK293 cells were transfected with plasmid expression vectors for WT or mutant MD-2, TLR4, CD14, *Firefly* luciferase NF-κB reporter, and *Renilla* luciferase control. Twenty-four hours after transfection, cells were treated with 10 μM heme or 10 ng/ml LPS for 6 h in media containing 0.1% serum. After 6 h, cells were lysed and luciferase activity was measured and expressed as the ratio of *Firefly* luciferase NF-κB reporter to *Renilla* luciferase control. Luciferase activity is expressed as a percent of cells transfected with WT MD-2 stimulated with heme or LPS (100% activity, black bars and horizontal dashed black line). Magenta cluster and green cluster refer to the 2 clusters of potential heme binding amino acids in Figure 3. Results are representative of 3 independent experiments run in triplicate. Bar values are means ± SD. **P < 0.01 vs. WT MD-2 using Kruskal-Wallis One Way Analysis of Variance on Ranks.

We next examined the effect of MD-2 point mutations on heme and LPS stimulation in the NF-κB reporter assay. MD-2 amino acids located at the potential heme binding sites on MD-2 identified in the magenta and green amino acid clusters in Figure 3 were each mutated individually to alanine. HEK293 cells were transfected with wild-type (WT) or mutant MD-2, WT CD14, WT TLR4, and the NF-κB luciferase reporter. NF-κB reporter activity was then expressed as a percent of WT MD-2 (100%) with heme or LPS stimulation (Figure 5). MD-2 point mutants W23A or Y34A in the magenta cluster significantly reduced heme-mediated NF-κB luciferase activity to 61 and 22%, respectively, relative to WT MD-2 (Figure 5, left). The S33A MD-2 mutation in the magenta cluster was similar to WT MD-2 NF-κB luciferase activity with heme stimulation. In contrast, MD-2 mutations in the green cluster, Y36A markedly stimulated heme-induced NF-κB luciferase activity to 191% compared to WT MD-2 (Figure 5, left). The C37A and I44A MD-2 mutations in the green cluster were not significantly different from WT MD-2. When the transfected HEK293 cells were stimulated with LPS (Figure 5, right), MD-2 mutations in the magenta and green cluster had no significant effects on NF-κB reporter activity relative to WT MD-2.

### Heme Binding to Mutant MD-2

**Figure 6:**
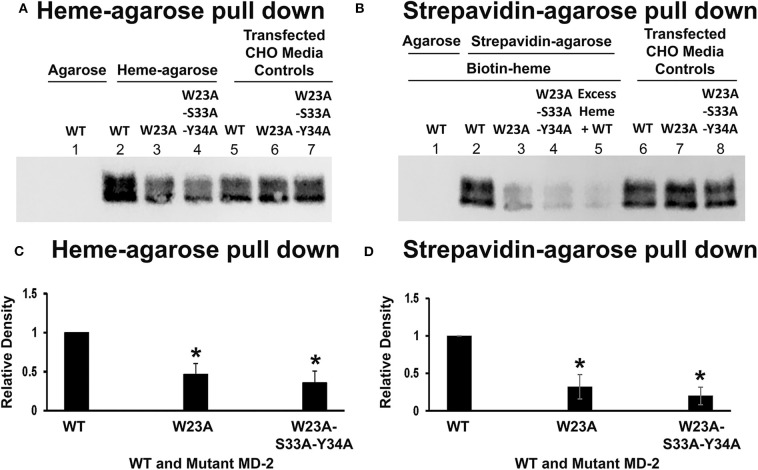
Heme binds poorly to W23A and W23A-S33A-Y34A mutant MD-2. WT MD-2, W23A MD-2, and W23A/S33A/Y34A MD-2 with Flag tags were expressed in CHO cells and the MD-2-containing media were used in heme-agarose and biotin-heme/streptavidin-agarose pull-down assays. Three days after transfection, the conditioned CHO media contained similar amounts of WT, W23A, and W23A/S33A/Y34A MD-2 on Western blots using Flag detection (**A**, lanes 5, 6, and 7 and **B**, lanes 6, 7, and 8). Heme-agarose pulled down only 47% of the W23A and 36% of the W23A/S33A/Y34A MD-2 mutants compared to WT MD-2 (**A**, lanes 2, 3, and 4 and **C**). WT MD-2 incubated with control agarose without heme was not pulled down (**A**, lane 1). Similarly, the biotin-heme/streptavidin-agarose pulled down only 32% of the W23A and 20% of the W23A/S33A/Y34A MD-2 mutants compared to WT MD-2 (**B**, lanes 2, 3, and 4 and **D**). WT MD-2 incubated with biotin-heme was not pulled down by agarose without streptavidin (**B**, lane 1). As previously shown, the addition of excess unlabeled heme markedly reduced the amount of WT MD-2 pulled down by biotin-heme/streptavidin-agarose (**B**, lane 5). The results shown in **(A,B)** are representative of four independent experiments. **(C,D)** show the results of quantitation of the Western blots. Bars are means + SD. *P < 0.05 compared to WT MD-2 using a student's unpaired *T*-test.

To determine if MD-2 mutations at the W23/S33/Y34 site affect heme binding to MD-2, WT MD-2, W23A MD-2, and W23A/S33A/Y34A MD-2 with Flag tags were expressed in CHO cells and the MD-2-containing media were used in heme-agarose and biotin-heme/streptavidin-agarose pull-down assays. Four days after transfection, the conditioned CHO media contained similar amounts of WT, W23A, and W23A-S33A-Y34A MD-2 on Western blots using Flag detection (Figure 6A, lanes 5, 6, and 7 and Figure 6B, lanes 6, 7, and 8). Heme-agarose pulled down 47% of the W23A and 36% of the W23A/S33A/Y34A MD-2 mutants compared to WT MD-2 (Figure 6A, lanes 2, 3, and 4 and Figure 6C). WT MD-2 was not pulled down from conditioned medium by control agarose without heme (Figure 6A, lane 1).

Similarly, the biotin-heme/streptavidin-agarose pulled down 32% of the W23A and 20% of the W23A/S33A/Y34A MD-2 mutants compared to WT MD-2 (Figure 6B, lanes 2, 3, and 4 and Figure 6D). WT MD-2 incubated with biotin-heme was not pulled down from conditioned medium by agarose without streptavidin (Figure 6B, lane 1). As previously shown, the addition of excess unlabeled heme markedly reduced the amount of WT MD-2 pulled down by biotin-heme/streptavidin-agarose (Figure 6B, lane 5).

## Discussion

Heme is essential for life, but when released from damaged cells it can act as a DAMP that promotes activation of MD-2/TLR4 signaling (7, 9, 12). Sickle cell disease (SCD), sepsis, malaria, viral hemorrhagic fevers, trauma, and hemorrhagic stroke can release large amounts of heme into the vasculature thereby promoting pro-inflammatory responses and tissue damage. We used pull-down assays and UV-VIS absorbance spectroscopy to demonstrate that recombinant MD-2 can bind heme. *In silico* analyses identified two potential heme docking sites on both human and murine MD-2 near conserved amino acids W23/S33/Y34 and Y36/C37/I44. HEK293 cells transfected with plasmids encoding WT MD-2, TLR4, and CD14 produced robust NF-κB reporter activity when stimulated with heme, LPS, or heme + LPS. NF-κB reporter activity was lost when MD-2, TLR4 or CD14 was omitted. MD-2 point mutations W23A and Y34A markedly inhibited the reporter responses to heme, but not LPS compared to WT MD-2. These data suggest that heme initiates MD-2/TLR4 signaling upon docking with MD-2 amino acids W23 and Y34. Y34 is a tyrosine residue; we speculate that the OH group on Y34 interacts with the iron moiety of heme as the heme iron is required for MD-2/TLR4 signaling (7, 9). W23 is a tryptophan which may provide a hydrophobic docking site for the vinyl groups on heme as the vinyl groups on heme are also required for MD-2/TLR4 signaling (7). The role of CD14 in heme-mediated MD-2/TLR4 signaling is unclear, but CD14 might be involved in the transfer of heme to MD-2 as has been reported for LPS (5, 19–21).

The function of sMD-2 has been extensively studied in LPS-TLR4 signaling. This LPS-sMD-2-TLR4 activation model is different from the classic LPS-TLR4 activation model in which MD-2 is co-localized with TLR4 on the cell surface, indispensable for LPS recognition and signaling (6). Heme-mediated MD-2/TLR4 signaling by macrophages does require MyD88 (7), suggesting dimerization of TLR4. However, it is possible that the model of a circulating sMD-2-heme complex to activate TLR4 is different from the model in which heme binds to the MD-2 co-localized with TLR4 on cell surface.

The activation of TLR4 signaling is unlikely to have been caused by LPS contamination of our hemin preparations. Endotoxin levels were monitored using a Limulus amebocyte lysate test. Heme preparations (10 mM) contained <0.01 endotoxin units/ml. Our NF-κB reporter assays used 10 uM hemin. We have previously shown that anti-LPS IgG directed against the LPS core does not inhibit heme-induced TLR4 signaling (9), but significantly inhibits LPS-induced MD-2/TLR4 signaling. The original paper describing the activation of TLR4 signaling by heme (7), showed that signaling of heme through TLR4 depended on an interaction distinct from the one established between TLR4 and lipopolysaccharide (LPS) since anti-TLR4/MD2 antibody or a lipid A antagonist inhibited LPS-induced TNF-alpha secretion but not heme activity. These data indicate that heme activates TLR4 signaling independently of LPS. In addition, the specificity of heme activation of MD-2/TLR4 signaling is supported in several published papers by our lab and others showing that the high affinity heme-binding protein, hemopexin, blocks the activation of MD-2/TLR4 signaling by heme (9, 14, 16, 22–24).

Some of our MD-2 Western blots appear to have 2 bands. Ohnishi et al. (25) reported that MD-2 has N-linked glycosylations at Asn (25) and Asn(114), which give up to 3 molecular weight bands on SDS PAGE gels. When cellular extracts are treated with N-glycosidase, only a single band with the fastest mobility was detected. It is likely the two bands seen on our MD-2 Western blots represent different glycosylation states of MD-2. Glycosylation of MD-2 appears to be important for TLR4-mediated signal transduction of LPS (25, 26). The impact on signal transduction of heme is unknown, but should be explored further.

There could potentially be other heme-activation sites on MD-2 or TLR4 that were not identified. We used a specialized algorithm that combines both structure- and sequence-based methods to identify potential heme-binding sites on heme proteins (17). This algorithm identified the same two distinct clusters of potential heme-binding amino acids at W23/S33/Y34 and Y36/C37/I44 on the surface of both human and mouse MD-2. Point mutations at W23A and Y34A inhibited heme activation of TLR4 signaling and a point mutation at W23A and a triple mutation at W23A-S33A-Y34A inhibited heme binding to MD-2. In contrast, point mutations S33A, Y36A, C37A, and I44A either stimulated or had little effect on heme-mediated MD-2/TLR4 signaling. Thus, it seems likely that the pocket located at W23 and Y34 on MD-2 is the most likely site for heme docking and activation of TLR4 signaling. However, a limitation of this study is the incomplete heme-binding data with the other MD-2 site mutations at S33A, Y34A, Y36A, C37A, and I44A. Confirmation of the NF-kB activation data in Figure 5 and the heme binding site on MD-2 awaits additional studies to confirm the W23/Y34 activation site.

Another possibility is that mutations of MD-2 at the proposed activation site might affect the folding and 3-dimensional structure of MD-2 and thereby indirectly affect MD-2 binding to heme and TLR4 signaling. However, nearby mutations Y36A, C37A, and I44A on MD-2 did not inhibit heme-mediated MD-2/TLR4 signaling. An important caveat of our data interpretation assumes the mutants were properly folded.

**Figure 7:**
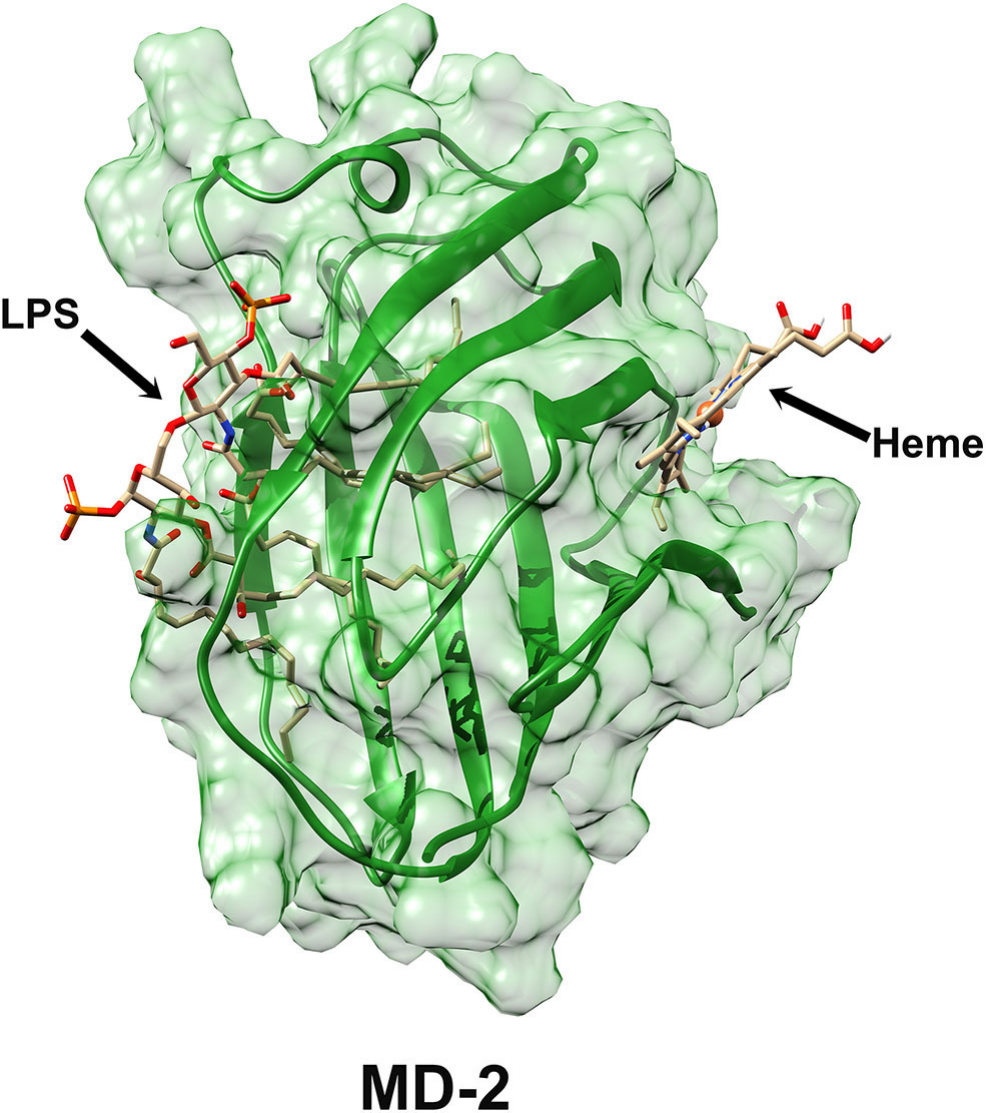
Surface representation of MD-2 structure derived from Protein Data Bank 3VQ2 (29) showing the LPS-binding site and the putative heme-binding site on MD-2.

Another limitation is the limited characterization of heme-MD-2 interactions. Future experiments will further characterize the interactions of heme with MD-2 using differential scanning fluorimetry to detect heme's binding affinity to MD-2 (27). Heme binding to MD-2 will be confirmed using secondary screens of melting by circular dichroism (non-fluorescent confirmation of ligand-binding stabilizing MD-2) and analytical centrifugation (non-fluorescent confirmation of heme-binding to MD-2 and stoichiometry). Heme-binding will also be analyzed by isothermal titration calorimetry. Our laboratory was unable to obtain reproducible data on heme binding to recombinant MD-2 using surface plasmon resonance. Verification of the heme docking site on MD-2 awaits confirmation of the crystal structure as was done with TLR4-MD-2 complexes with LPS and LPS analogs (6, 28–30). The interaction with LPS is mediated by a hydrophobic internal pocket in MD-2. However, there appears to be no overlap of MD-2 residues involved in the binding of LPS and the MD-2 heme-binding site at W23 and Y34 near the N-terminus. In fact, the heme and LPS binding sites are located on opposite sides of the MD-2 protein (Figure 7), which might partially explain why mutations at the heme binding site had no significant effects on the LPS-stimulated NF-κB reporter assays.

Future studies will use virtual screening to identify small molecules that might interact at the W23/Y34 site and inhibit heme-mediated MD-2/TLR4 signaling. Their interaction with MD-2 will be characterized by differential scanning fluorimetry, circular dichroism, and analytical centrifugation as described above for heme-MD-2 interactions. Candidate molecules will be screened for their ability to inhibit heme-mediated chemokine production in human monocyte/macrophages. Follow-up screens will include inhibition of heme activation of P-selectin and von Willebrand factor expression on the membrane of endothelial cells, vaso-occlusion in SCD mice, and heme binding to MD-2.

We conclude that heme activates TLR4 signaling at residues W23 and Y34 on MD-2. This site on MD-2 appears to be a possible target for inhibition of heme-mediated TLR4 signaling. We speculate that targeted inhibition of heme-mediated TLR4 signaling would be beneficial in hemolytic diseases such as SCD without affecting innate immunity to gram negative bacteria expressing LPS.

## Data Availability Statement

All datasets generated for this study are included in the article/Supplementary Material.

## Author Contributions

JB and GV designed the research. JT and JH contributed computational tools. JB, PZ, KN, JT, and GV wrote and edited the manuscript. JB, PZ, and JT prepared the figures. PZ, JN, and ZK performed the biochemical analyses. All authors contributed to the article and approved the submitted version.

## Conflict of Interest

The authors declare that the research was conducted in the absence of any commercial or financial relationships that could be construed as a potential conflict of interest.
