# Supplementary material for: Identification of a Heme Activation Site on the MD-2/TLR4 Complex
Source: Front Immunol. 2020 Jun 30;11:1370. doi: 10.3389/fimmu.2020.01370 (PMC7338675; doi:10.3389/fimmu.2020.01370)
Supplement: Supplementary file 1 [file Data_Sheet_1.docx]

**SUPPLEMENTAL MATERIAL**

**Identification of a Heme Activation Site on the MD-2/TLR4 Complex**

John D. Belcher^*^, Ping Zhang, Julia Nguyen, Zachary M. Kiser, Karl A. Nath, Jianjun Hu,
John O. Trent, Gregory M. Vercellotti

**
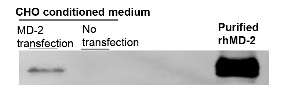
**

**Supplemental Figure 1.** **Transfected CHO cells secrete soluble recombinant human MD-2 into the media.** CHO cells were transfected with N-flag-tagged human MD-2 DNA and then incubated in protein-free media for 72 hours. The resulting CHO media and N-flag-tagged recombinant human MD-2 (rhMD-2) purified from the transfected CHO media were run on a Western blot with detection using a primary antibody to MD-2.


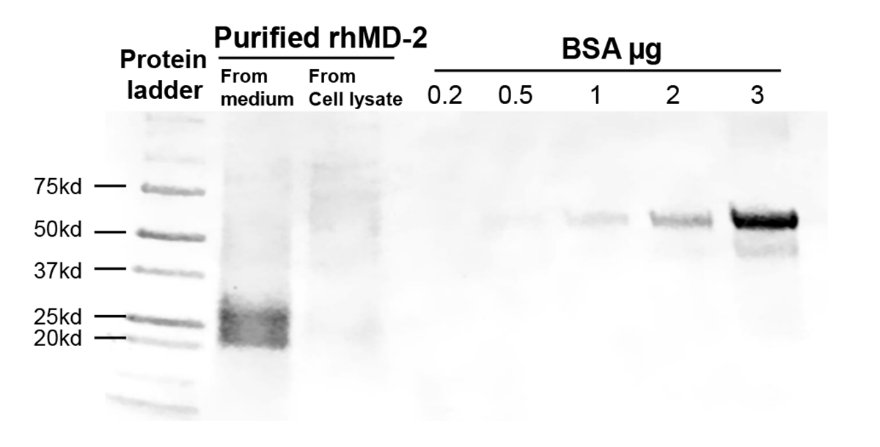


**Supplemental Figure 2. Recombinant human MD-2 (rhMD-2) is highly purified from transfected CHO medium.** N-flag-tagged rhMD-2 was purified from the media and cell lysate of CHO cells transfected with MD-2 DNA using anti-flag affinity chromatography**.** The resulting rhMD-2 preparations were run on an SDS PAGE gel and stained with Coomassie Brilliant Blue R-250. Bovine serum albumin (BSA) was loaded onto the gel at different protein levels (0.2 – 3 µg), so that the concentration of rhMD-2 could be estimated by comparing it with the intensity of the BSA protein bands.
